# Newborn Neurobehavior Is Related to Later Neurodevelopment and Social Cognition Skills in Extremely Preterm-Born Children: A Prospective Longitudinal Cohort Study

**DOI:** 10.3389/fpsyg.2021.710430

**Published:** 2021-09-06

**Authors:** Leena Aho, Marjo Metsäranta, Piia Lönnberg, Elina Wolford, Aulikki Lano

**Affiliations:** ^1^Department of Paediatrics, Children's Hospital, Pediatric Research Center, University of Helsinki and Helsinki University Hospital, Helsinki, Finland; ^2^Department of Psychology and Logopedics, University of Helsinki, Helsinki, Finland

**Keywords:** alertness, preterm birth, neonatal orientation, neurodevelopment, social cognition, visuomotor

## Abstract

**Objective:** The aim of this study was to evaluate the ability of the neonatal neurobehavioral characteristics to act as an indicator for later neurodevelopment and neurocognitive performance.

**Methods:** Sixty-six infants born extremely preterm (<28 gestational weeks) were followed until 6.5 years. Neurobehavior at term age was assessed by the behavior subscale of the Hammersmith Neonatal Neurological Examination (HNNE) using dichotomic rating, optimal, and non-optimal. The Griffiths Mental Developmental Scales (GMDS) at 2 years, and the Wechsler Intelligence Scales at 6.5 years, and a Neuropsychological Assessment at 6.5 years were used to assess neurodevelopment and neurocognitive performance including social cognition skills.

**Results:** An optimal auditory orientation at term age was associated with better developmental quotients (DQ) in Personal–Social, and Hearing–Language GMDS subscale at 2 years (*p* < 0.05). An optimal visual alertness was associated with better Total (*p* < 0.01), Locomotor (*p* < 0.001), and Eye–Hand Coordination (*p* < 0.01) DQs at 2 years, and with sensorimotor function (*p* < 0.001) and social perception (*p* < 0.01) tests at 6.5 years.

**Conclusion:** The neurobehavioral characteristics of newborns might serve as a precursor of social cognition skills and the HNNE behavior subscale offers a tool to identify infants at risk for later deficits in neurodevelopment and social cognition.

## Introduction

Extremely preterm-born children (EPT) are at high risk for sensorimotor, language, and visuocognitive impairments, deficits in attention and executive function, and poor academic achievement (Geldof et al., 2012; Johnson and Marlow, 2014). In addition, they are subject to poor social competence comprising social cognition, social interaction, and social adjustment (Ritchie et al., 2015; Taylor, 2020). Social cognition, a rarely investigated domain in preterm cohorts, consists of the mental processes used to assess and interpret both verbal and non-verbal social cues such as facial expressions and body movements to understand others in social interaction (Wocadlo and Rieger, 2006; Williamson and Jakobson, 2014; Taylor, 2020). These abilities are essential for flexible adaptation to variable social contexts and demands and have been suggested to play an important role in social development (Williamson and Jakobson, 2014). There is evidence that very low birth weight children have problems not only in recognizing facial emotion expressions, but also more broadly in social perception predisposing them for social and behavioral impairments (Wocadlo and Rieger, 2006; Williamson and Jakobson, 2014).

Understanding of neonatal predictors of childhood outcomes is important for early detection of neurodevelopmental impairments and for allocating timely interventions at an early age of the potential brain plasticity (Cioni et al., 2011). Thus, there is a need for sensitive, reliable, and non-invasive instruments suitable for clinical routine to identify children at-risk.

Neonatal neurological assessments at term age, while emphasizing the newborn neuromotor repertoire, also provide tools to identify the deficits in the sensory and behavioral function of infants (Wusthoff, 2013). The Hammersmith Neonatal Neurological Examination (HNNE) is a reliable assessment that can be easily integrated into the standard follow-up care (Noble and Boyd, 2012). The HNNE behavior subscale includes tasks of auditory and visual orientation, alertness, irritability, consolability, and cry. Decreased capacity to attend to visual and auditory stimuli, higher excitability, poorer self-regulation, and more difficulty in consoling compared with full-term infants are neurobehavioral deficits observed in very preterm infants at term age (Brown et al., 2006). Visual following and auditory orienting at term age have been suggested to offer a tool to assess the initial capacities of neonates (Wallace et al., 1995). Moreover, neonatal behavioral characteristics, such as orientation, irritability, and self-regulation have been related to both short- and long-term cognitive development and intelligence (Lundqvist-Persson, 2001; Canals et al., 2011). To our knowledge, there are no studies demonstrating associations between the neurobehavioral characteristics of newborns and social cognition skills at pre-school age. We have earlier shown a relationship between visual fixation with sustained alertness at term age and visuomotor performance at 2 years in our longitudinal study cohort of EPT children (Stjerna et al., 2015). The primary objective of this study was to test in the same cohort whether the neurobehavioral characteristics of newborns using HNNE act as an indicator of neurocognitive function and social cognition skills of later childhood.

## Materials and Methods

### Participants

**Figure 1:**
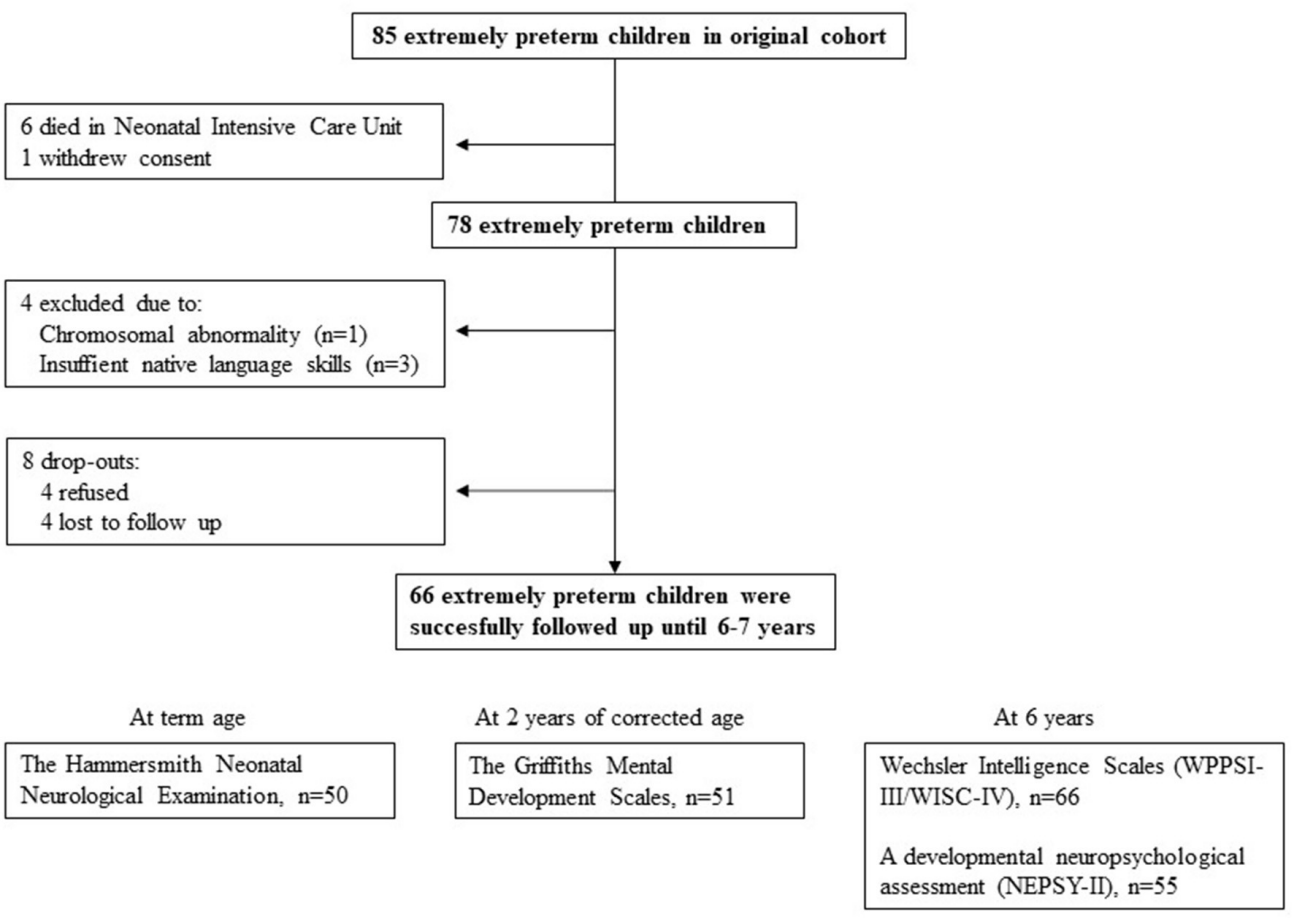
Study flowchart and dropouts.

The original cohort of this longitudinal multimethodological prospective study included 85 EPT infants who were born before 28 gestational weeks and were actively treated after birth at the neonatal intensive care unit of the Helsinki University Hospital, Finland, between May 2006 and September 2008 (KeKeKe study—Extremely Preterm Birth and Development of the Central Nervous System). The present study included 66 children who had been successfully followed until 6.5 years of age, as shown in Figure 1. The characteristics of the participated and dropout children are shown in Table 1. The original study and the follow-up at 6.5 years were approved by the Ethics Committee for gynecology and obstetrics, pediatrics, and psychiatry of the Hospital District of Helsinki and Uusimaa. The written informed consent to participation and publication of the results was obtained from parents or guardians. In addition, all children received age-appropriate information about the study and provided consent to participate in the study.

**Table 1:** Characteristics of the study population.

	**Extremely preterm-born children *n* = 66**	**Extremely preterm-born dropouts *n* = 12**	***p***
Boys	43 (65%)	6 (50%)	<0.001
Gestational age, mean (SD)	26,4 (1.2)	26,3 (1.2)	0.93
Birth weight, g mean (SD)	858 (187)	918 (113)	0.17
Twins	16 (24%)	0 (0%)	0.11
Small for gestational age, SD < −2,0	8 (12%)	1 (8.3%)	1.0
**Neonatal morbidity**			
Prenatal corticosteroids	65 (98%)	12 (100%)	1.0
Respiratory distress syndrome	49 (74%)	6 (50%)	0.17
Bronchopulmonary dysplasia at 36+0 gestation weeks	32 (51%)	2 (17%)	0.06
Necrotizing enterocolitis	5 (7.5%)	2 (17%)	0.52
Patent ductus arteriosus	52 (79%)	10 (83%)	1.0
Sepsis	32 (51%)	5 (42%)	0.76
Retinopathy of prematurity	22 (34%)	2 (17%)	0.170
**Intraventicular hemorrage**			0.35
No	39 (59%)	9 (75%)	
Grades I–II	15 (31%)	3 (25%)	
Grades III–IV	12 (10%)		
**White matter injury in MRI at term age**			0.03
Normal	36 (59%)	8 (90%)	
Mild	19 (31%)	1 (10%)	
Moderate	6 (10%)		
**Mother's education**			0.03
High school or lower	30 (48%)	1 (12%)	
Bachelor degree	18 (29%)	4 (44%)	
Master degree or higher	14 (23%)	4 (44%)	
**Father's education**			0.10
High school or lower	33 (55%)	2 (25%)	
Bachelor degree	18 (30%)	3 (37.5%)	
Master degree or higher	9 (15%)	3 (37.5%)	

*Data that are not available for EPT children are as follows: Bronchopulmonary dysplasia 3, retinopathy of prematurity 1, MRI 5, educational level of mothers 4, educational level of fathers 6; for dropout EPT children: Bronchopulmonary dysplasia 1, MRI 3, educational level of mothers 3, educational level of fathers 4*.

*Fisher's exact, Mann-Whitney U, and Kendall's tau B tests were used*.

### Clinical Data

Obstetric and neonatal data were collected from the medical records and are presented in Table 1. Socioeconomic data were obtained from parental questionnaires. Gestational age was based on the first-trimester ultrasound. Small for gestational age was defined as a birth weight below −2.0 SD for gestational age based on the Finnish growth reference data. Bronchopulmonary dysplasia was defined as a need for additional oxygen at 36 + 0 weeks of gestational age. Retinopathy of premature was diagnosed by an ophthalmologist at routine visits. Severe retinopathy was defined as ROP Stage III treated with retinal laser photocoagulation. Patent ductus arteriosus was determined by cardiac ultrasound. The highest grade of intraventricular hemorrhage in serial cranial ultrasound was recorded. A MRI was performed at the term age. White matter injury was classified into four categories from none to severe and gray matter injury was categorized into three groups (Woodward et al., 2006).

### Neonatal Neurological Assessment

The neonatal neurological examination was performed by an experienced pediatric neurologist (AL) using standardized HNNE. In the present study, we selected five from the six items of HNNE behavior subscale including auditory orientation, visual orientation, alertness, irritability, and consolability. It has been demonstrated that the behavior subscale shows little variation with gestational age (Dubowitz et al., 1998). We used dichotomic rating, optimal, and non-optimal. The following performances were defined as optimal: “shifting of eyes, head might turn toward source” and “prolonged head turn to stimulus, search with eyes, smooth” for the auditory orientation, “following horizontally and vertically and turning head,” and “following in a circle” for the visual orientation, “keeps interest in stimuli” for visual alertness, “awakes, cries sometimes when handled” for irritability, and “infant cries; becomes quiet when talked to” for consolability. We used the standard black and white bull's eye target for assessing visual orientation and visual alertness.

### Neurodevelopmental Assessment at 2 Years of Corrected Age

The Griffiths Mental Developmental Scales (GMDS) was used to assess neurodevelopment at 2 years of corrected age. The Griffiths scales provide an overall developmental quotient (DQ) based on five subscales: Locomotor, Personal-Social, Hearing-Language, Eye-Hand Coordination, and Performance. An English normative sample was used as a reference (mean overall DQ 100.5, SD 11.8) (Huntley, 1996). The severe delay was defined as DQ below −2 SD, a mild delay as DQ between −2 SD and −1 SD, and DQ over −1 SD (88.7) was considered average.

### Neuropsychological Assessment at 6.5 Years of Age

The neuropsychological assessment at 6.5 years of age consisted of the Finnish edition of the Wechsler Preschool and Primary Scale of Intelligence—Third Edition (WPPSI-III) or the Wechsler Intelligence Scale for Children—Fourth Edition (WISC-IV), as well as the Finnish edition of NEPSY-II, a neuropsychological test battery (Korkman et al., 2008a; Wechsler, 2009, 2010). Full Scale (FSIQ), Verbal (VIQ), and Performance (PIQ) Intelligence Quotients (mean 100, SD 15) were derived from three Performance (Block Design, Matrix Reasoning, and Picture Completion) and two Verbal (Information and Vocabulary) subtests of the WPPSI-III or the WISC-IV. Twelve sub-tests were used from the NEPSY-II to test different areas of neuropsychological development: executive functioning/attention (Auditory attention, Visual attention), memory and learning (Memory for designs, Memory for faces, Narrative memory), sensorimotor functioning (Imitating hand positions), visuospatial processing (Arrows, Block construction, Design copying, Geometric puzzles), and social perception (Affect recognition, Theory of mind). Age-adjusted standard scores for the subtests were calculated according to the Finnish norms (mean 10, SD 3; scores 1–3 well below expected level, scores 4–5 below expected level, scores 6–7 borderline, scores 8–12 at expected level, and scores 13–19 above expected level) (Korkman et al., 2008b).

### Statistical Analysis

Statistical analysis was conducted using SPSS for Windows program version 25. Background variables (as shown in Table 1) between participating EPT and dropout children and between EPT children with the optimal and non-optimal performance on the HNNE were compared using Fisher's exact test and Kendall's tau B test for categorical variables and Mann–Whitney *U*-test and Kruskal–Wallis test for continuous variables. Non-parametric Mann–Whitney *U*-test or Kruskal–Wallis test when appropriated were used to evaluate DQs in GMDS at 2 years and standard score in NEPSY at 6.5 years as continuous variables according to neonatal morbidity factors (as shown in Table 1), educational level of mothers, and the HNNE outcome (categorical). Furthermore, an independent samples *t*-test for normally distributed continuous variables was used to evaluate WPPSI-III/WISC-IV IQs at the age of 6.5 years with neonatal morbidity factors, educational level of mothers, and the HNNE outcome. The univariate linear model was performed to study the relationship between the performance on the HNNE subscales and outcome variables: DQs in GMDS, FIQ, VIQ, and PIQ and NEPSY standard scores. Results are reported as coefficients B and their 95% confidence interval (CI). The adjusted linear regression models did not fulfill the model assumptions, and the residuals were not normally distributed and could not be applied. All significance tests were two-tailed and *p* < 0.05 was considered statistically significant.

## Results

The characteristics of the study population are presented in Table 1. The group of dropouts was considerably smaller than the group of participating EPT children (12 vs. 66). There were significantly more boys and the educational level of mothers was lower among participating EPT children compared with dropout EPT children. None of the children had severe white matter injury at term age. Participating EPT children had more often mild-to-moderate white matter injury compared with dropout children. Six of the participating EPT children and none of the dropouts had severe retinopathy.

### The HNNE Behavior Assessment

At term age (range: 38–45 weeks of gestational age), 50 EPT children were assessed using the HNNE. Boys more often had non-optimal visual alertness and consolability compared with girls (*p* = 0.016 and *p* = 0.034, respectively). In all other items of behavior subscale, boys and girls succeeded equally. The neonatal morbidity factors (Table 1) were not significantly related to the performance on assessed items of the HNNE.

### Neurodevelopmental and Neuropsychological Outcome

Table 2 shows the results for the GMDS, WPPSI-III or WISC-IV, and NEPSY-II.

**Table 2:** The results of neurodevelopmental and neuropsychological tests at 2 and 6.5 years of age.

		**n**	**Mean**	**SD**
**The Griffiths Mental Developmental Scales, DQ (mean 100.5, SD 11.8)**				
Total		51	86.7	10.9
Locomotor		51	85.5	11.2
Personal-Social		51	86.1	11.6
Hearing-Language		51	83.9	16.4
Eye-hand Coordination		51	87.6	11.5
Performance		51	90.2	9.6
**Intelligence Scales: WPPSI-III or WISC-IV, IQ (mean 100, SD 15)**				
FSIQ		66	91.6	16.6
VIQ		66	96.2	21.5
PIQ		66	87.0	16.3
**NEPSY-II, standard score (mean 10, SD 3)**				
Executive function/attention	Auditory attention	48	9.0	2.4
	Visual attention	48	9.6	1.9
Memory and learning	Memory for designs	47	8.8	3.0
	Memory for faces	46	9.0	2.4
	Narrative memory	55	9.4	2.9
Sensorimotor function	Imitating hand positions	47	7.0	3.2
Visuospatial processing	Arrows	50	9.3	2.3
	Block construction	51	9.6	3.1
	Design copying	54	7.3	1.9
	Geometric puzzles	47	9.7	2.9
Social perception	Affect recognition	49	9.2	2.9
	Theory of mind	48	9.1	2.4

*DQ, developmental quotient; WPPSI-III, wechsler preschool and primary scale of intelligence—third edition; WISC-IV, wechsler intelligence scale for children—fourth edition; FSIQ, full scale; VIQ, verbal, PIQ, performance intelligence quotients; NEPSY-II, a developmental neuropsychological assessment*.

At the corrected age of 2 years (mean 24.9 months, range: 23–27 months), six (9%) EPT children had cerebral palsy. None of the children were blind or deaf. Fifty-one EPT children were assessed using GMDS. Overall, 14% of the EPT children had severe neurodevelopmental delay, and 29% had mild delay. Higher educational level of mothers was significantly associated with better DQs in Locomotor, Personal-Social, and Hearing-Language subscales. The retinopathy of prematurity was the only neonatal morbidity factor that significantly impaired the performance in all other GMDS subscales except Locomotor (*p* < 0.05).

A total of 66 children were assessed using WPPSI-III or WISC-IV at 6.5 years. Notably, 12.1% of the children had FSIQ scores <70 indicating severe delay, and 12.1% had scores below average (70–84) indicating mild delay. The neonatal morbidity factors (Table 1) were not significantly related to the performance on WPPSI-III or WISC-IV. Higher educational level of mothers is associated significantly with better FSIQs and VIQs (*p* < 0.05). Fifty-five children were assessed using NEPSY-II. The standard scores in Imitating hand positions and Design copying were borderline. In all other subtests, standard scores were in the average range.

### The Associations Between HNNE Behavior Subscale and Later Neurodevelopmental Outcome and Neuropsychological Performance

**Figure 2:**
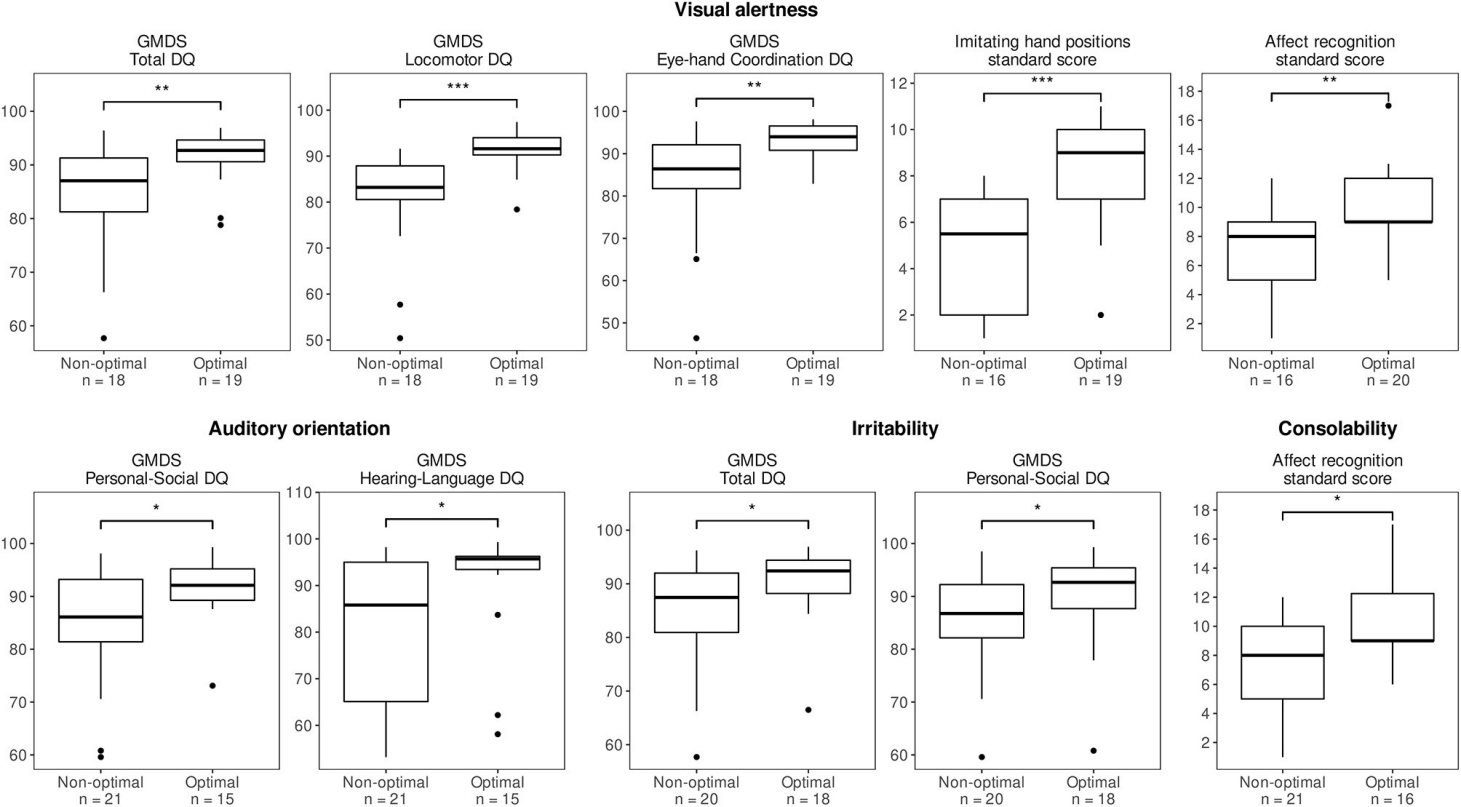
The relationships between the performance on the Hammersmith Neonatal Neurological Examination (HNNE) items and the Griffiths Mental Developmental Scales (GMDS) and a Developmental Neuropsychological Assessment. Boxplots represent median, lower, and upper quartiles. Mann-Whitney *U*-test was used, and only significant findings are presented. *p < 0.05, **p < 0.01, ***p < 0.001.

Table 3 and Figure 2 show the significant associations between the performance on the HNNE at term age and the neurodevelopmental/neuropsychological assessments at 2 and 6.5 years. An optimal auditory orientation was associated with better DQs in Personal-Social, and Hearing-Language subscale (*p* < 0.05) at 2 years. There was no association between auditory orientation and neuropsychological performance at 6.5 years; however, there was a trend that children with an optimal auditory orientation had higher verbal IQ (103 vs. 95, *p* = 0.15). The performance in the visual orientation at term age was not associated with neurodevelopmental outcome and neuropsychological performance at 2 and 6.5 years.

**Table 3:** Associations between the performance on the HNNE behavior items and DQs in GMDS and standard scores in NEPSY-II.

**HNNE items**	**Optimal/non-optimal n**			**n**	**Coeff B**	**95% CI**	***p***
Auditory orientation	19/29	GMDS	Personal-Social	36	7.3	0.84–13.7	0.028
			Hearing-Language	36	10.4	0.06–20.7	0.049
Visual orientation	15/34		No significant associations				
Visual alertness	24/25	GMDS	Total	37	7.9	2.39–13.5	0.006
			Locomotor	37	10.6	5.15–16.1	<0.001
			Eye-Hand Coordination	37	9.2	2.80–15.7	0.006
		NEPSY-II	Imitating hand positions	35	3.7	1.84–5,53	<0.001
			Affect recognition	36	3.1	1.19–4.96	0.002
Irritability	23/27		No significant associations				
Consolability	20/30	NEPSY-II	Affect recognition	37	2.4	0.49–4.39	0.015

*HNNE, the hammersmith neonatal neurological examination; GMDS, the griffiths mental developmental scales; DQ, developmental quotient; NEPSY-II, a developmental neuropsychological assessment. The univariate linear regression model was performed and only the statistically significant findings are presented, p < 0.05. Coeff, Coefficients B (mean difference)*.

An optimal visual alertness was associated with better DQs in Total (*p* < 0.01), Locomotor (*p* < 0.001), and Eye-Hand Coordination subscale (*p* < 0.01) at 2 years. There was no association between visual alertness and scores in Intelligence Scales (WPPSI-III or WISC-IV) at 6.5 years. Children with optimal visual alertness compared with children with non-optimal performance scored 3.7 standard scores (~1 SD) more in sensorimotor function (CI 1.8–5.5, *p* < 0.001) and 3.1 standard scores (~1 SD) more in Social perception test, Affect recognition (CI 1.2–5.0, *p* = 0.002) at 6.5 years of age.

Children with an optimal performance on the HNNE irritability got higher mean DQs in Total and Personal-Social subscale than children with non-optimal performance (*p* < 0.05). Comparison of GMDS and Intelligence Scales (WPPSI-III and WISC-IV) results showed no significant differences between children who performed optimally vs. non-optimally on the HNNE consolability. However, children with optimal compared with non-optimal consolability at term age attained 2.4 standard scores more in Social perception test, Affect recognition (CI 0.5–4.4, *p* = 0.015).

## Discussion

Our aims were to determine whether the neurobehavioral performance of EPT infants, reflecting the higher-order functioning and regulatory capacities of neonates, may be used to predict later neurocognitive outcome and social cognition skills, and to identify in clinical routine the infants who are at a neurodevelopmental risk.

Expanding our previous study of the neurobehavioral characteristics of newborns (Stjerna et al., 2015) from visual abilities to the other items of the HNNE behavior subscale and extending the follow-up of EPT children up to the age of 6 years, we observed that in EPT infants, visual alertness at term age was significantly associated with sensorimotor function, imitating hand positions, and social cognition traits, precisely affect recognition. Furthermore, auditory orientation was associated with language and social development at 2 years of corrected age. In addition, consolability showed a significant relationship with affect recognition indicating better social cognition skills. For the first time, we were able to show that visual alertness is associated not only with visual cognitive development, but also with the development of social cognition skills.

In agreement with an earlier study (Wallace et al., 1995), we found that optimal auditory orientation at term age-predicted good performance on Personal-Social, and Hearing-Language subscale at corrected age of 2 years. Furthermore, children with optimal auditory orientation had higher verbal IQs, although not statistically significant, at 6.5 years suggesting continuity from early infancy to preschool age. Caregiver-infant mutual gaze interaction lays the foundation for joint attention, where eye gaze serves as an important cue to guide the attention of infants (Grossmann and Johnson, 2007; Hoehl et al., 2009). The ability of infants to joint attention is an important part of the process through which children learn the language. Preterm children have problems already in this preverbal stage showing less initiating or responding to joint attention (De Schuymer et al., 2011). It seems that preverbal skills serve as a link between preterm birth and language development (De Schuymer et al., 2011). Infants who show better orientation to novel visual and auditory stimuli may be more responsive to the environment, and this ability is a key prerequisite of learning (Wallace et al., 1995; Kushnerenko et al., 2002).

The preferential attention of newborns to human faces and eyes immediately after birth plays a crucial role in early social and cognitive development (Hoehl et al., 2009). It has been suggested that the amygdala, which is important for emotion recognition, takes part in orientating the attention of infants toward faces (Leppänen and Nelson, 2006). We found that EPT children with non-optimal visual alertness at term perform worse than the children with optimal visual alertness in the affect recognition test, assessing emotions from facial expression. Very low birth weight children have difficulty in interpreting the emotions of others, mainly due to a failure to identify key non-verbal cues from facial and body movements (Williamson and Jakobson, 2014). Williamson and Jakobsen suggested that the difficulties primarily reflect a visual processing deficit. However, initially, infants use more multimodal cues, i.e., synchronous facial and vocal stimuli, to detect emotional expression (Leppänen and Nelson, 2009). In line with this, we found that poor auditory orientation to stimuli and poor visual alertness at term age predicted deficits in social cognition skills.

We showed that optimal visual alertness neonatally was related to a better score in the sensorimotor function test, imitating hand positions at 6.5 years of age. The imitation of meaningless gestures is a complex task requiring visuomotor processes, visual attention, and working memory (Goldenberg, 2001; Rumiati et al., 2005). The EPT children also performed below average in a visuomotor Design copying test. Functioning in both tests relies on the dorsal stream, connecting the occipital and posterior parietal cortices, especially vulnerable to prematurity (Atkinson, 2017). Prematurity may alter white matter tracts and reduce the surface area of visual-perceptual cortical regions (Sripada et al., 2015). Sensory experience of the neonatal intensive care environment may affect the development of the sensory system and together with prematurity fundamentally change the complex development of dorsal streams causing persistent visuomotor integration deficits (Sripada et al., 2015). To summarize, visual alertness at term age reflects integrity in subcortical networks, and later higher cognitive function is built up on the same networks (Johnson, 2005).

We observed that less irritable children got higher GMDS Total scores and Personal-Social scores at 2 years, but there was no association with the neuropsychological test results at 6.5 years. Instead, good consolability was related to better recognition of emotions from facial expressions at 6.5 years. General irritability and consolability are the characteristics of the self-regulation of infants. Our finding is in line with earlier reports that an infant with a low level of self-regulation is at risk for the poor development of social interaction (Lundqvist-Persson, 2001; Canals et al., 2011). At term age, good consolability and minor irritability predict better self-regulation, and further better self-regulation correlates with better attention regulation, one of the cornerstones of human cognition (Feldman, 2009). Moreover, attention networks overlap with extended dorsal streams, and, thus, EPT children are at risk for attention problems (Atkinson, 2017). EPT children with poor consolability together with poor visual alertness at term age are likely to have attention and social cognition problems later in life.

The strength of this study is the use of a longitudinal prospective design. The study cohort was homogeneous. None of the EPT children needed additional oxygen at term age indicating that BPD has already resolved, and none of the children had severe white matter injury in MRI. One of the strengths is wide cognitive testing including neuropsychological assessment and tests of social cognition at 6.5 years.

The main limitation of the study is the small number of EPT children and the lack of control subjects. Due to the longitudinal nature of the study, loss to follow-up is inevitable. It is possible that dropout children would have developed better than participating EPT children. There were more boys, the educational level of mothers was lower, and participating EPT children more often had white matter injury and ROP than dropouts, which may indicate that they are at a higher risk for neurodevelopmental problems than dropouts. It may be that dropout children would have performed better already at term age and also at 2 and 6.5 years, and, thus it is unlikely that they would remove the predictive value of the HNNE for later sensorimotor and social abilities. Some of the EPT children were unable to complete the test battery because of being in an inappropriate state for assessments, e.g., restless or due to attentional problems. Our cohort included high-risk EPT children limiting us to make conclusions only on EPT children. The educational level of mothers did not influence the NEPSY-II test results. Wocadlo and Rieger (2006) demonstrated that maternal social skills are more influential on the socioemotional development of children than any family factor including parental education. Generally, boys are more vulnerable for deficits in social skills (Fabes and Eisenberg, 1998) than girls and, thus, the bigger proportion of boys in our cohort may overestimate the problems. With a larger sample size, it would have been possible to find stronger associations and to use more sophisticated statistical methods to investigate the effect of confounding variables on the results.

## Conclusion

Our study demonstrated that the neurobehavioral characteristics of newborns at term age in EPT infants were related to neurodevelopmental outcome and neuropsychological performance at 2 years and at pre-school age. We were able to show that visual alertness is associated not only with visual cognitive development, but also with development of social cognition skills. Accordingly, the assessment of the neurobehavioral characteristics of newborns may offer a usable tool to identify infants at risk for later impairments and to enable timely consultation to promote sensitive parent-infant interaction, and the ability of parents to interpret the cues of their infants to support the early development and behavior of infants (Boykydis, 2008).

## Data Availability Statement

The raw data supporting the conclusions of this article will be made available by the authors, without undue reservation.

## Ethics Statement

The studies involving human participants were reviewed and approved by The Ethics Committee for gynecology and obstetrics, pediatrics, and psychiatry of the Hospital District of Helsinki and Uusimaa, Finland. Written informed consent to participate in this study was provided by the participants' legal guardian/next of kin.

## Author Contributions

MM, AL, and LA contributed to the study concept and design and interpretation of the data. AL, PL, and EW participated in the acquisition of data. LA carried out the analyses and drafted the initial manuscript. All authors revised the manuscript critically and approved the submitted final version of the manuscript. All the listed authors meet the appropriate authorship and substantially contributed to the study.

## Conflict of Interest

The authors declare that the research was conducted in the absence of any commercial or financial relationships that could be construed as a potential conflict of interest.

## Publisher's Note

All claims expressed in this article are solely those of the authors and do not necessarily represent those of their affiliated organizations, or those of the publisher, the editors and the reviewers. Any product that may be evaluated in this article, or claim that may be made by its manufacturer, is not guaranteed or endorsed by the publisher.
